# Supplementary figures and images for: Performance determinants of unsupervised clustering methods for microbiome data
Source: Microbiome. 2022 Feb 5;10:25. doi: 10.1186/s40168-021-01199-3 (PMC8817542; doi:10.1186/s40168-021-01199-3)

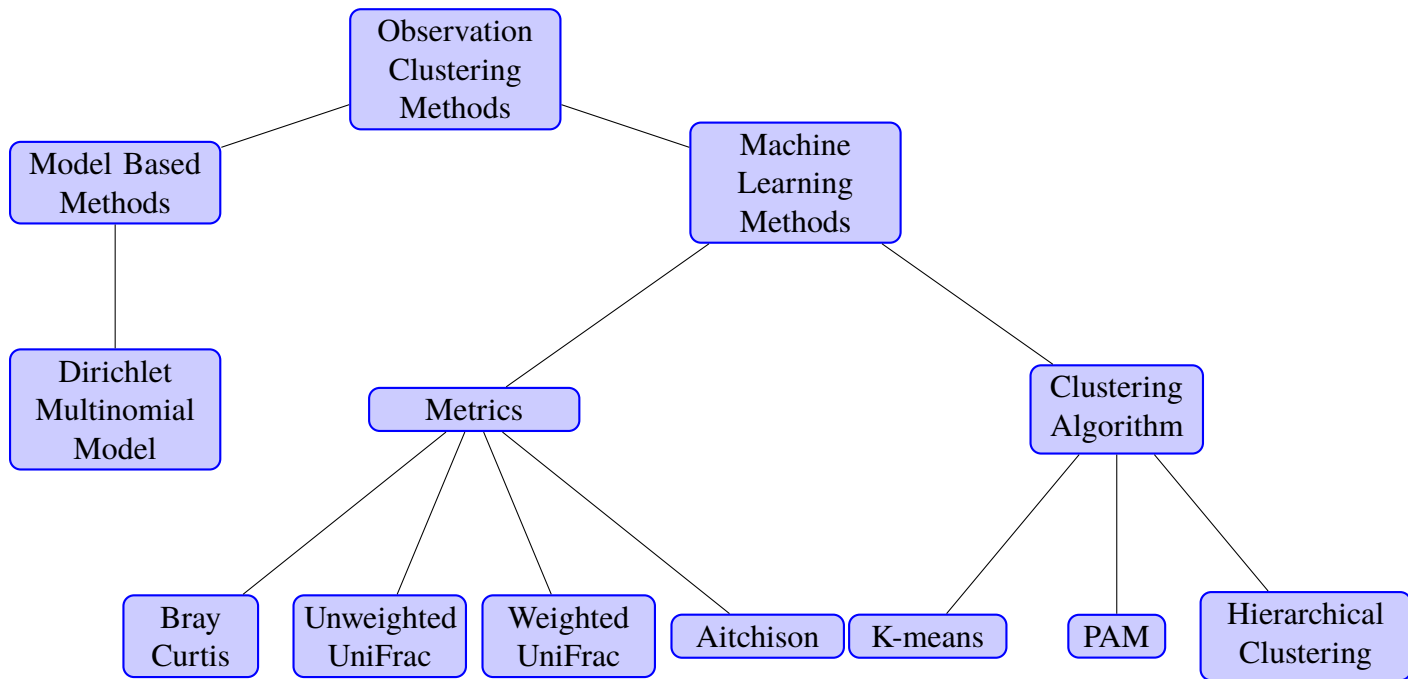

Supplement: Supplementary file 2 — Additional file 1 Figure S1. An illustrative plot of commonly used clustering methods. [file 40168_2021_1199_MOESM2_ESM.pdf]

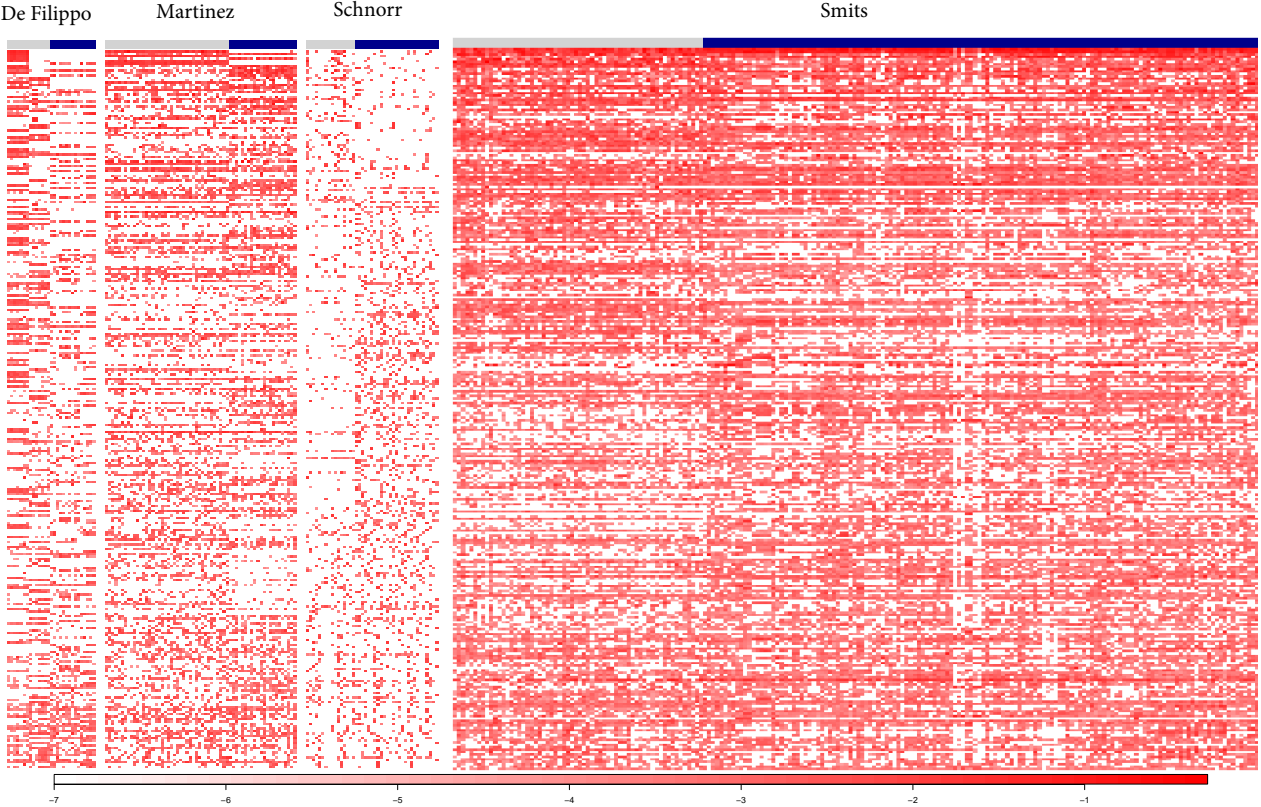

Supplement: Supplementary file 3 — Additional file 2 Figure S2. Heatmap of the most abundant 300 OTUs for the four example datasets. This figure shows the most abundant 300 OTUs for the four published datasets plotted in log 10 scale. Red color indicates high abundance, whereas white color indicates low abundance. As shown in the plot, the abundances of “high abundance” OTUs in Schnorr dataset are lower than those of the other three datasets. Gray and blue indicate different clusters in each dataset. [file 40168_2021_1199_MOESM3_ESM.pdf]

0 Levels Trimmed off

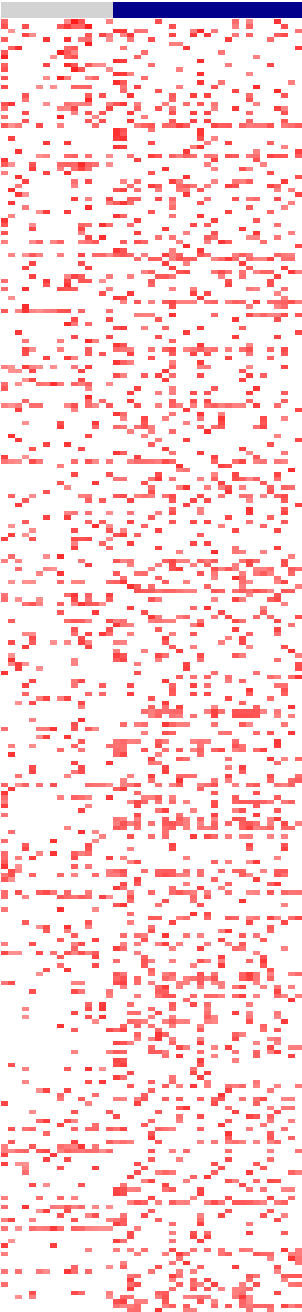

19 Levels Trimmed off

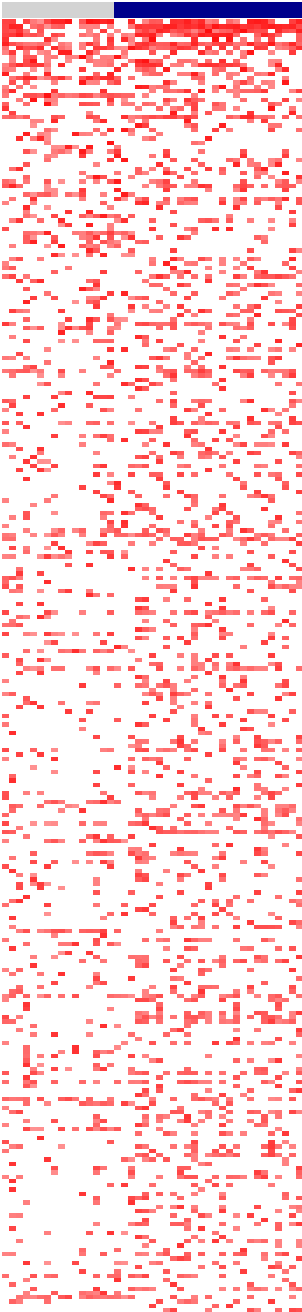

29 Levels Trimmed off

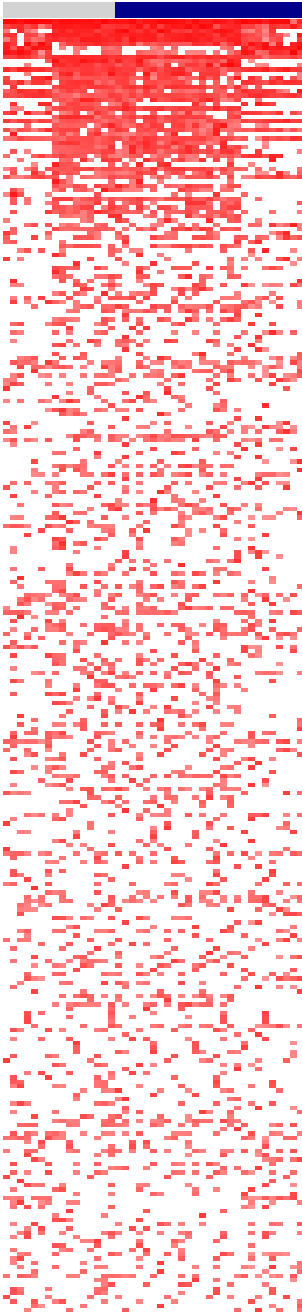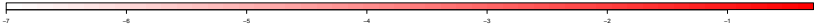

Supplement: Supplementary file 4 — Additional file 3 Figure S3. Heatmap of the most abundant 300 OTUs for the Schnorr dataset with 0, 19, and 29 levels trimmed off. This figure plots the most abundant 300 OTUs of the Schnorr Dataset with 0, 19, and 29 levels trimmed off. As trimming goes along, the abundant OTUs aggregate sequences from distant OTUs. In each subpanel, the gray left part is the Italian sample set, while the blue right part is the Hadza sample set. [file 40168_2021_1199_MOESM4_ESM.pdf]

Original

First Generation

Second Generation

Third Generation

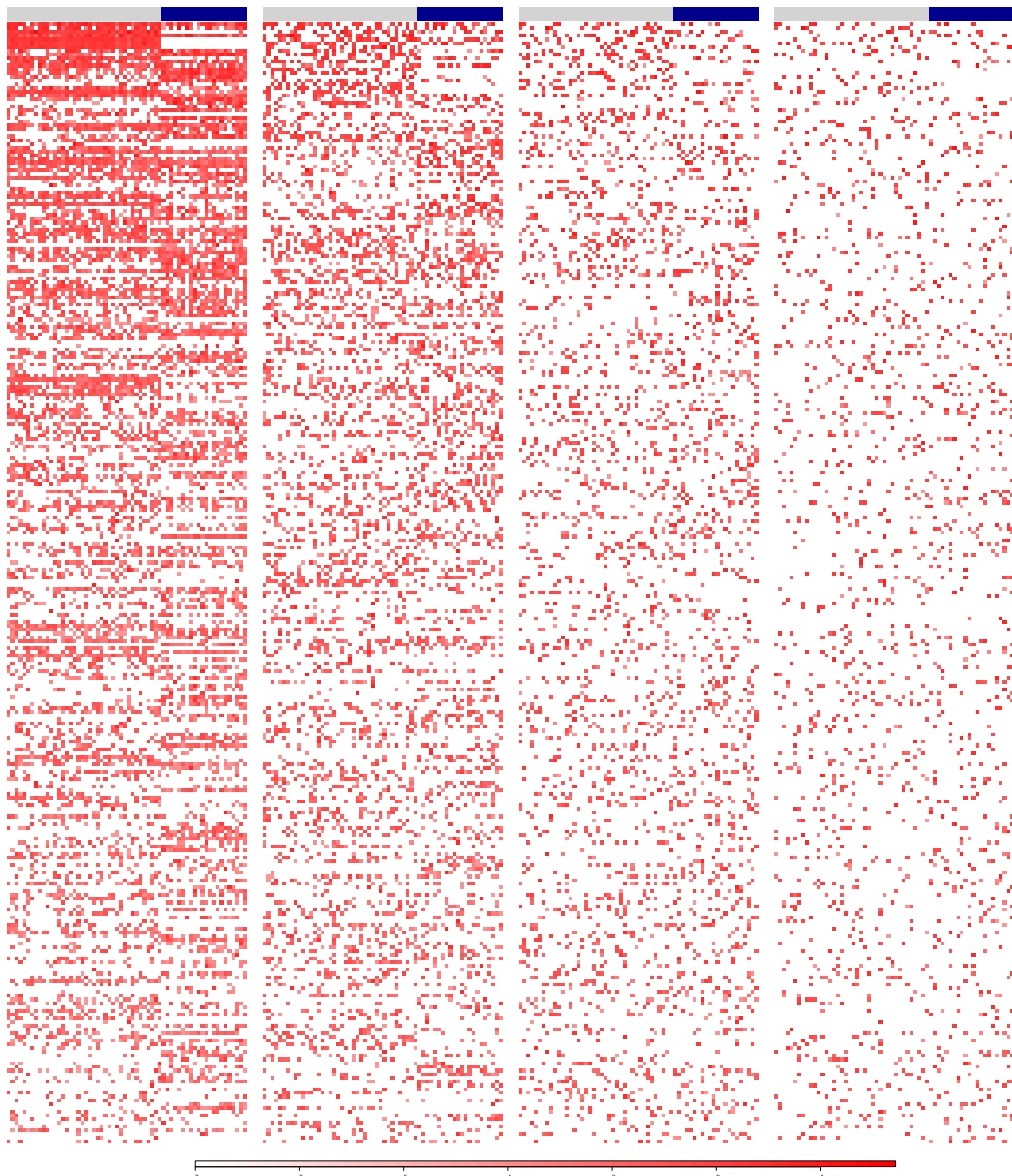

Supplement: Supplementary file 5 — Additional file 4 Figure S4. Heatmap of the most abundance 300 OTUs of the Martínez dataset, with descendants. This figure plots the most abundance 300 OTUs of the Martínez Dataset with its first, second and third generation descendants. As the tree branches diverge, fewer sequences are left in the most abundant 300 OTUs. In each subpanel, the gray left part is the Papua sample set, while the blue right part is the US sample set. [file 40168_2021_1199_MOESM5_ESM.pdf]

(A)

### Shannon

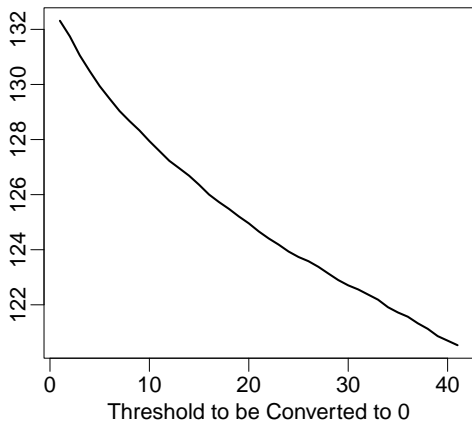

(B)

### Unweighted UniFrac performance

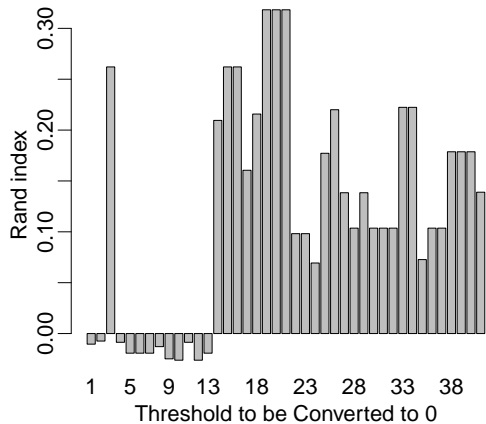

Supplement: Supplementary file 6 — Additional file 5 Figure S5. Coverting low abundance OTUs to 0s improves the performance of UU for Gopalakrishnan dataset. (A) Shannon diversity of the dataset decreases as more OTUs are converted to 0s. (B) The trend of the Rand index rises by converting OTUs to 0s, however excessive count removal affects the performance of UU eventually. [file 40168_2021_1199_MOESM6_ESM.pdf]

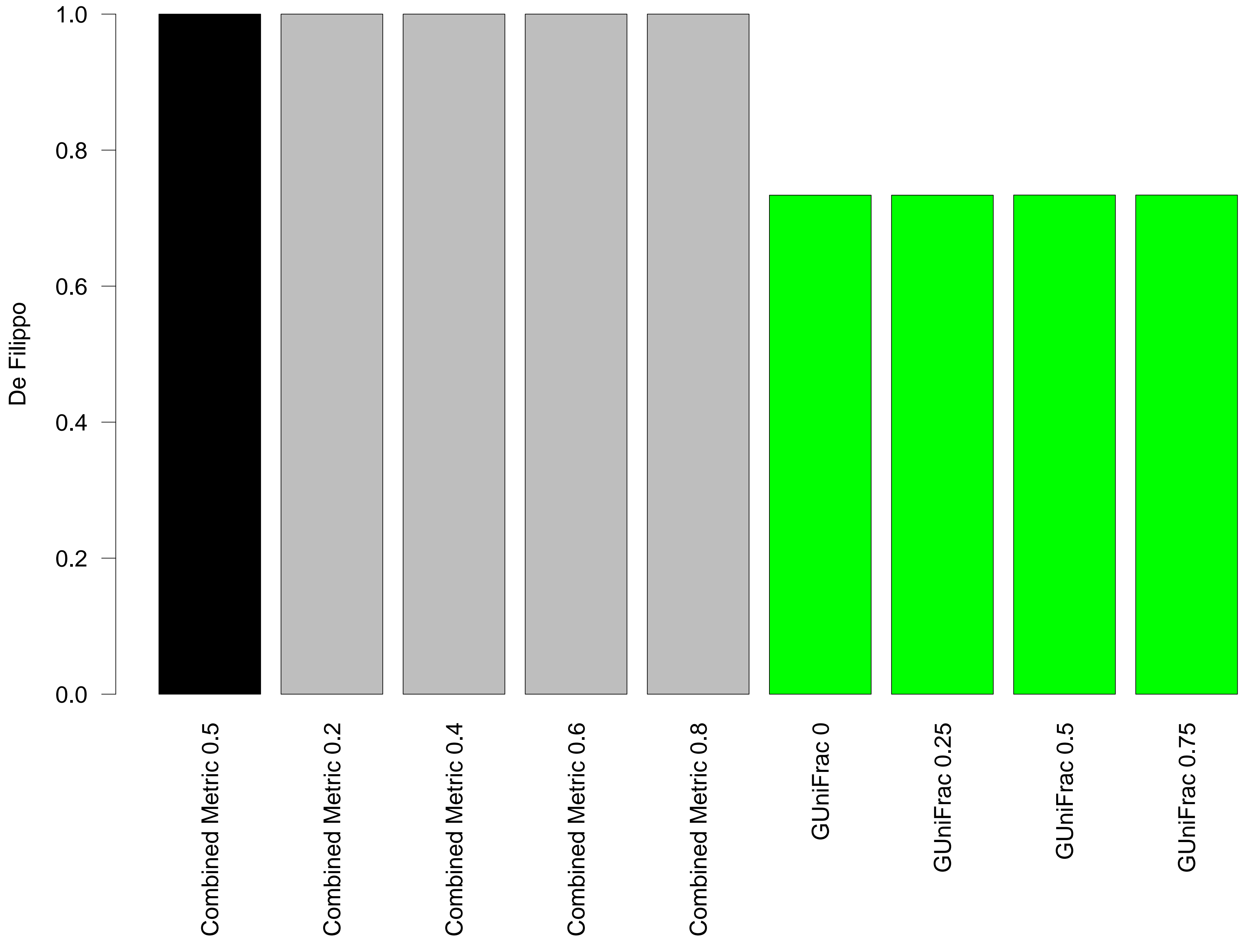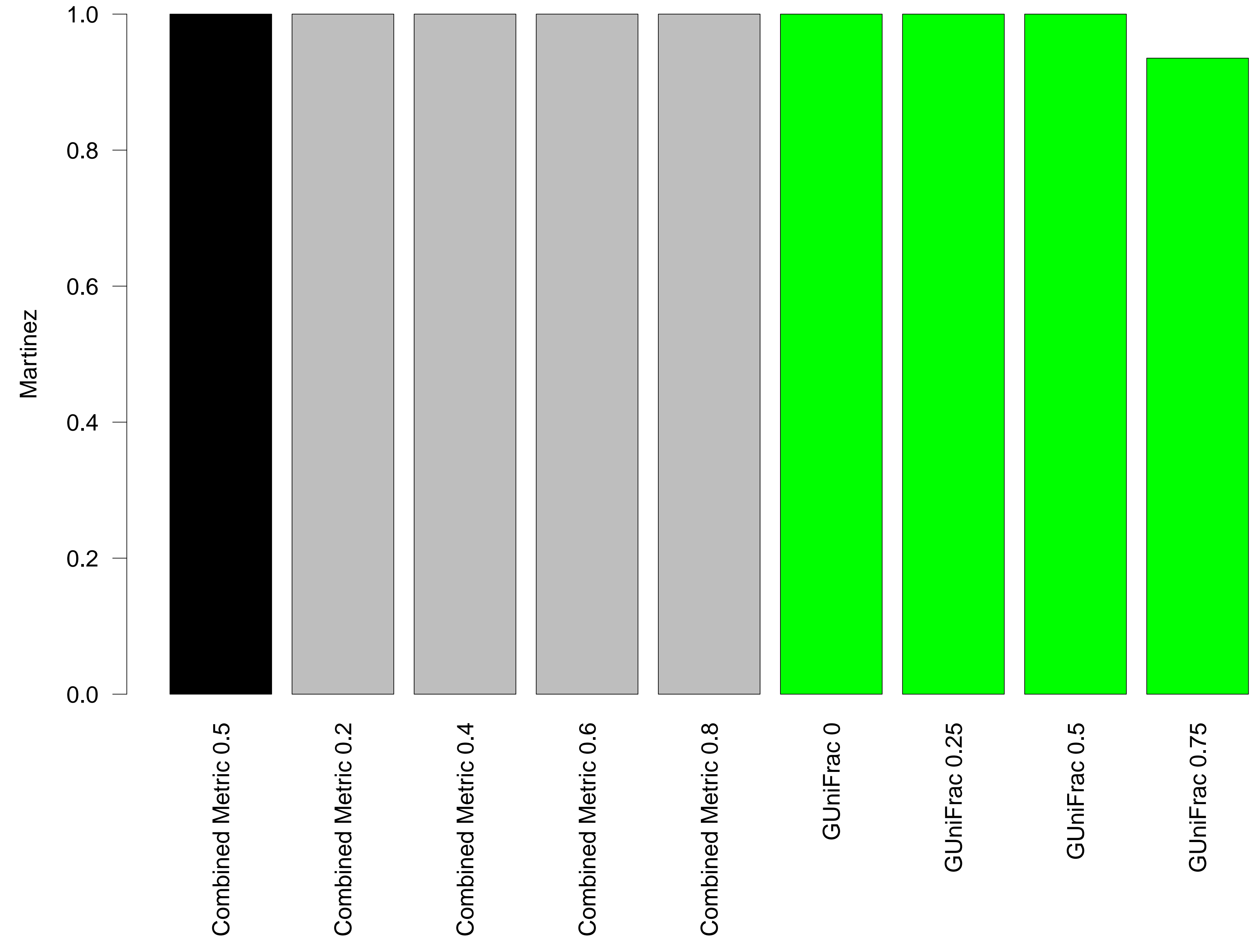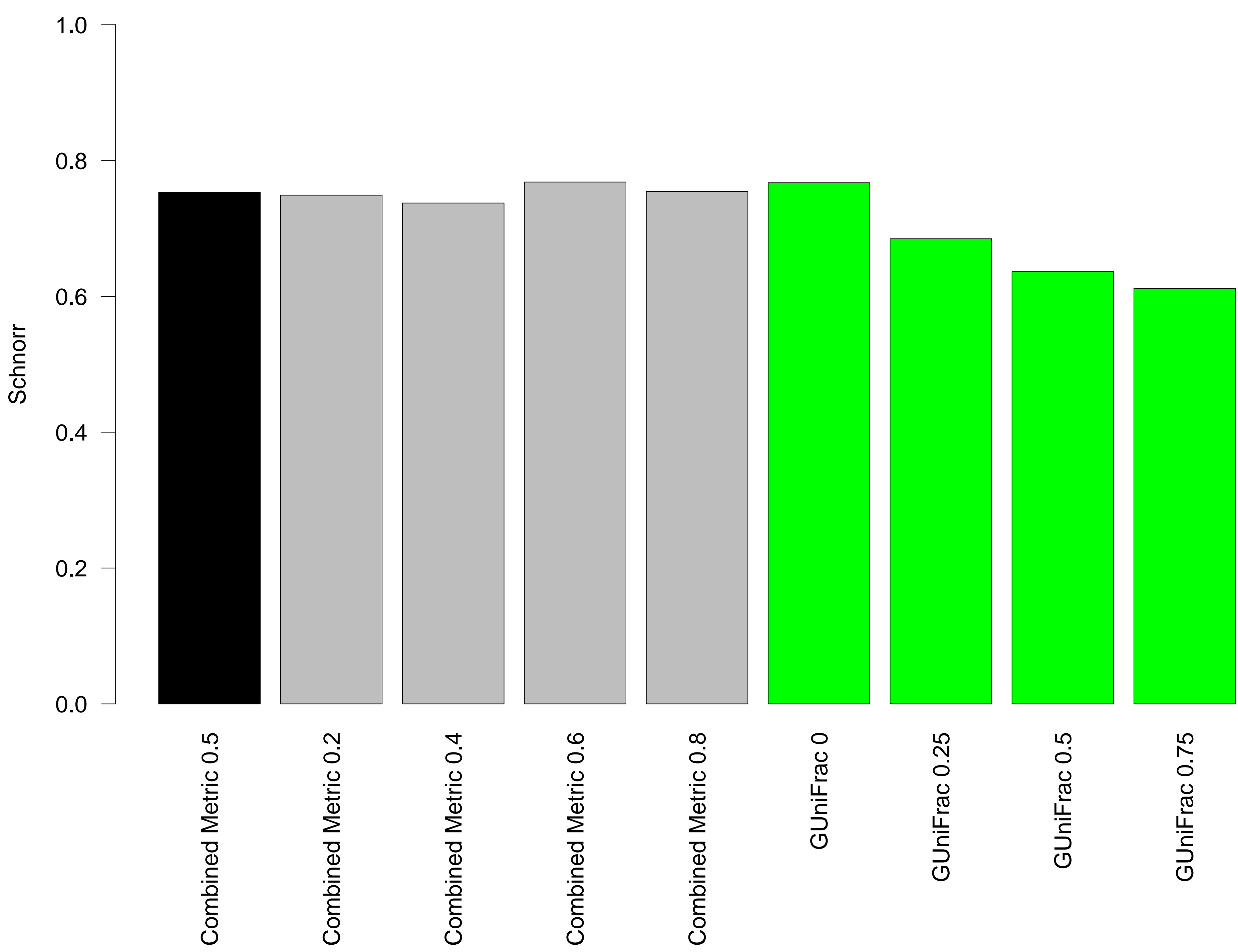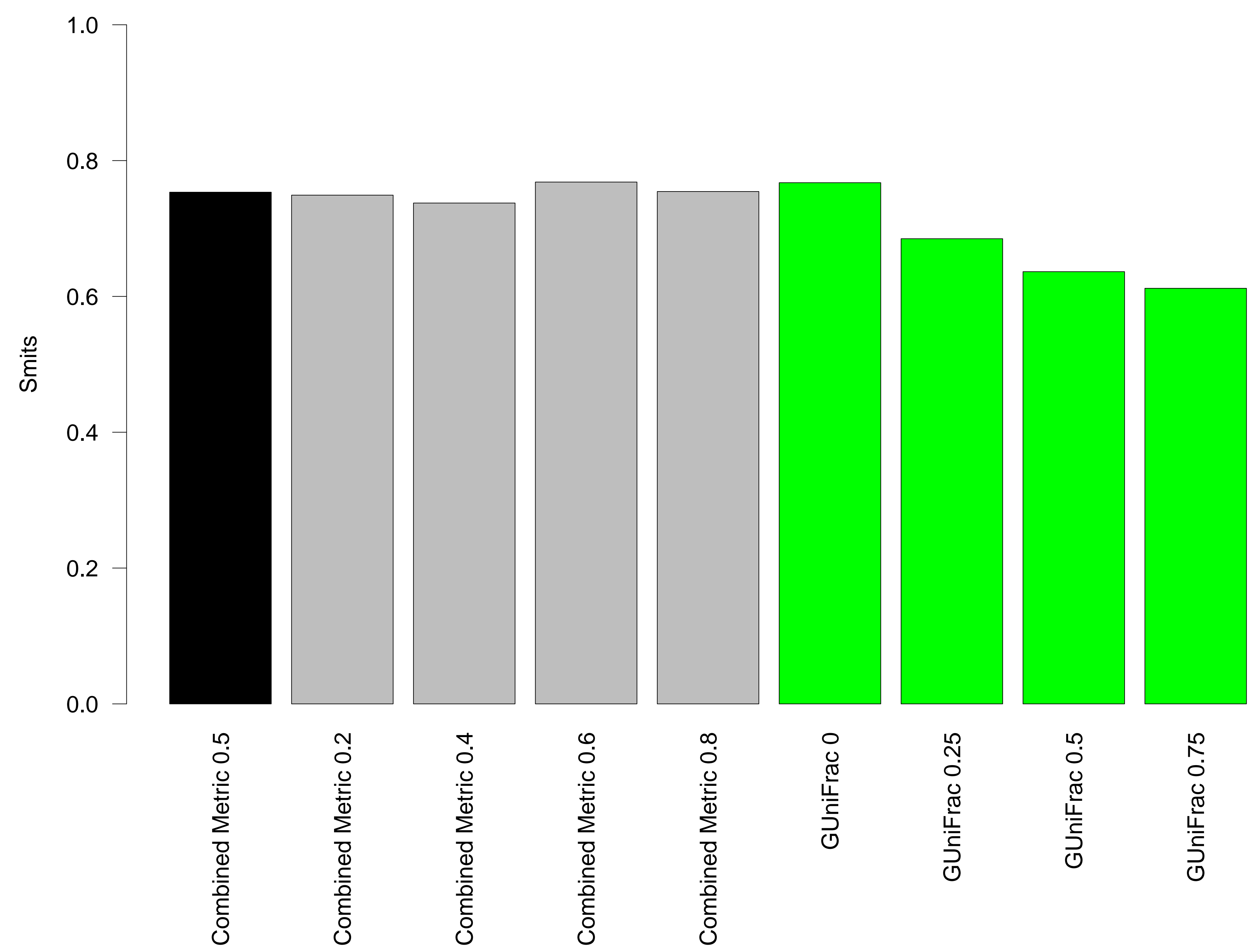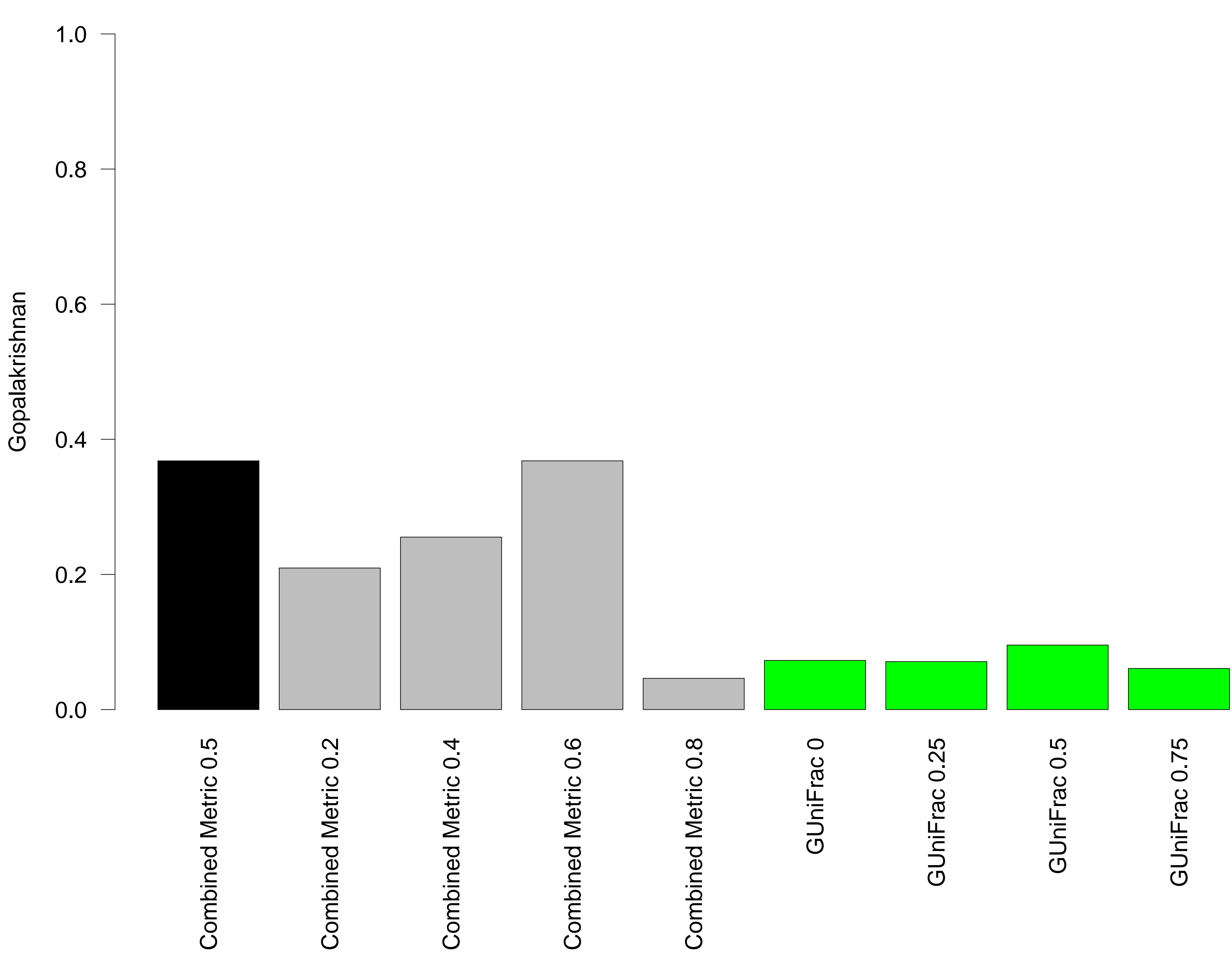

Supplement: Supplementary file 7 — Additional file 6 Figure S6. Rand indices with different α values for the proposed metric and comparison with the generalized UniFrac metric. This figure shows the Rand indices with different values of α (0.2, 0.4, 0.6, 0.8) for the proposed metric and compares the results with the Generalized UniFrac under different parameters for it (0, 0.25, 0.5, 0.75). [file 40168_2021_1199_MOESM7_ESM.pdf]

**PAM-Bray Curtis** **PAM-Unweighted Unifrac** **PAM-Weighted Unifrac** **PAM-Aitchison** **PAM-DMM**

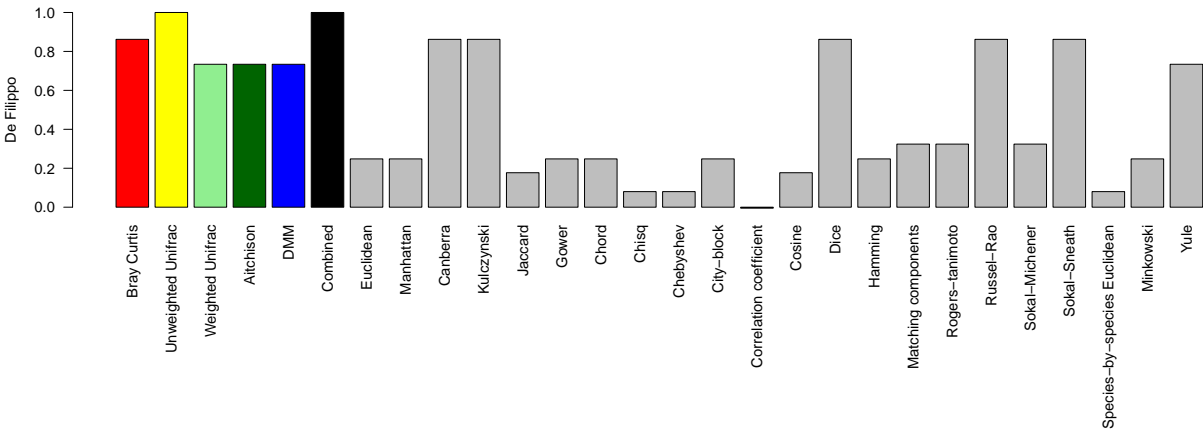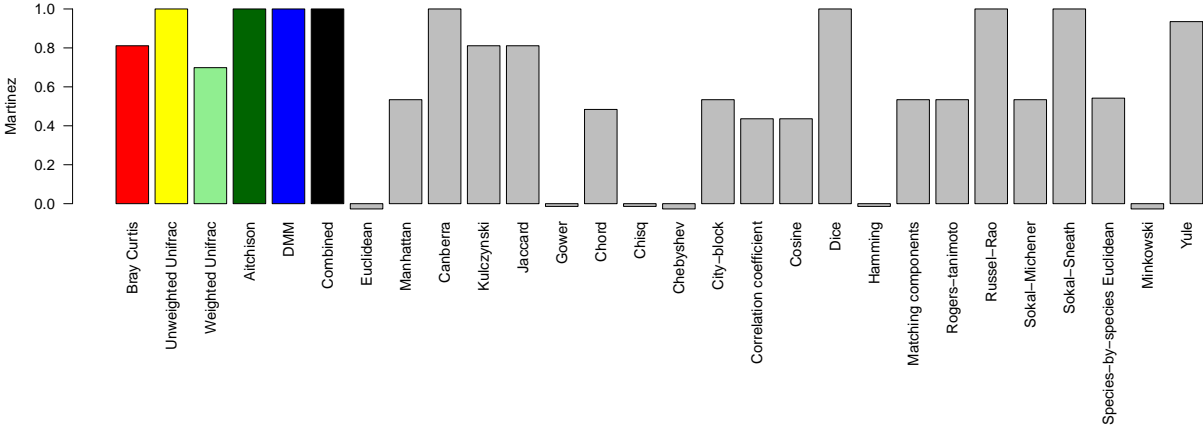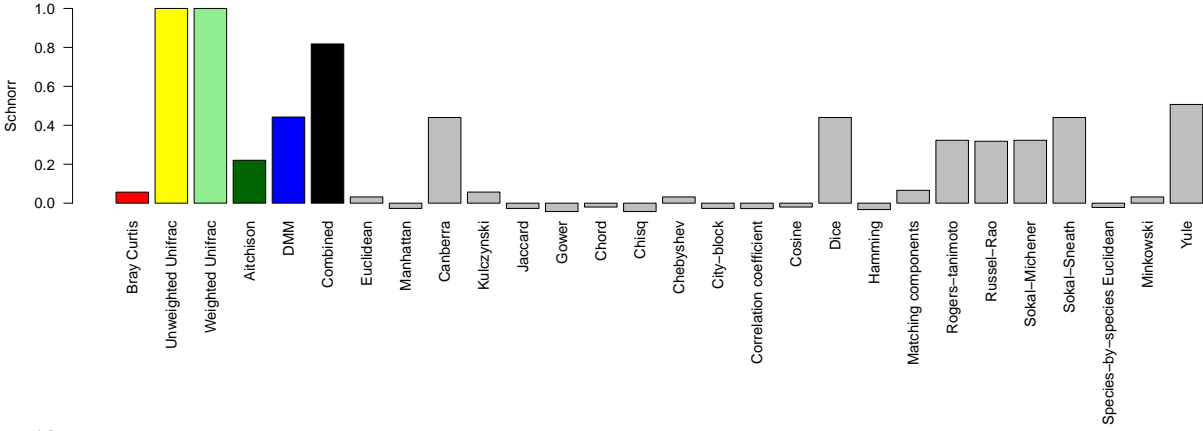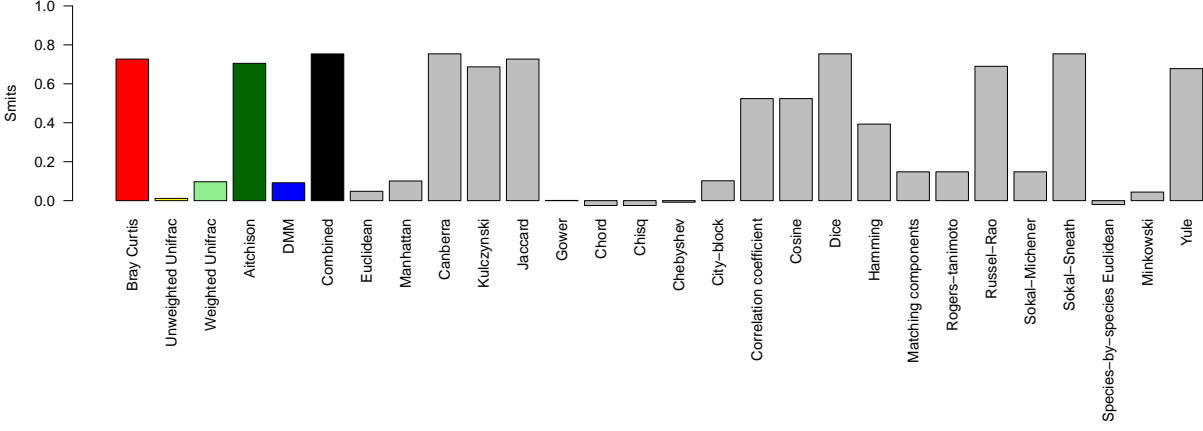

Supplement: Supplementary file 8 — Additional file 7 Figure S7. Performance of other metrics. The figure shows the performance of other less common metrics provided by QIIME2. Among them, city-block distance and species-by-species Euclidean distance are modifications of the Euclidean distance, while Jaccard distance and Canberra distances are similar to the Bray Curtis distance. As pointed out in the vegan package manual [27], Euclidean and Manhattan distances are not good in separating groups. More details of beta diversity metrics can be found in QIIME2 [23]. [file 40168_2021_1199_MOESM8_ESM.pdf]
