# Supplementary material for: Performance determinants of unsupervised clustering methods for microbiome data
Source: Microbiome. 2022 Feb 5;10:25. doi: 10.1186/s40168-021-01199-3 (PMC8817542; doi:10.1186/s40168-021-01199-3)
Supplement: Supplementary file 9 — Additional file 8 Table S1. Additional summary of the example datasets. [file 40168_2021_1199_MOESM9_ESM.pdf]

**Table S1 Additional summary of the example datasets**

| Dataset | Cluster | Skewness | Kurtosis | Percent of 0 Entries |
|---|---|---|---|---|
| De Filippo | | 6.39 | 72.19 | 77.2 |
| | Italy | 5.61 | 49.38 | |
| | Africa | 8.77 | 128.53 | |
| Martínez | | 23.91 | 780.56 | 83.2 |
| | Papua | 27.44 | 961.11 | |
| | US | 20.45 | 513.49 | |
| Schnorr | | 3.49 | 27.13 | 88.2 |
| | Hadza | 2.52 | 15.12 | |
| | Italy | 5.50 | 62.75 | |
| Smits | | 63.23 | 4649.79 | 85.7 |
| | Late Dry | 65.11 | 4805.62 | |
| | Early Wet | 37.40 | 1778.04 | |

The third and fourth columns of this table refer to the skewness and kurtosis of the averaged abundance vector of the whole dataset or each cluster. Skewness is often used as a measure of symmetry, while kurtosis is a measure for the degree of tailedness in the distribution.
